# Semi-artificial mouse skin membrane feeding technique for adult tick, *Haemaphysalis longicornis*

**DOI:** 10.1186/1756-3305-5-263

**Published:** 2012-11-15

**Authors:** Takeshi Hatta, Takeharu Miyoshi, Makoto Matsubayashi, Md Khyrul Islam, M Abdul Alim, Anisuzzaman, Kayoko Yamaji, Kozo Fujisaki, Naotoshi Tsuji

**Affiliations:** 1 Laboratory of Parasitic Diseases, National Institute of Animal Health, National Agricultural and Food Research Organization, 3-1-5 Kannondai, Tsukuba, Ibaraki, 305-0856, Japan; 2 Laboratory of Emerging Infectious Diseases, School of Frontier Veterinary Medicine, Kagoshima University, Korimoto, Kagoshima, 890-0065, Japan

**Keywords:** *Haemaphysalis longicornis*, Mouse skin membrane system, Semi-artificial feeding technique, Tick

## Abstract

**Background:**

An *in vitro* artificial feeding technique for hard ticks is quite useful for studying the tick-pathogen interactions. Here, we report a novel semi-artificial feeding technique for the adult parthenogenetic tick, *Haemaphysalis longicornis*, using mouse skin membrane.

**Findings:**

Skin with attached adult ticks was removed from the mouse body at 4 to 5 days post-infestation for the construction of the feeding system. This system supplied with rabbit blood was kept in >95% relative humidity at 30°C during the feeding, and ticks were fully engorged (artificially engorged, AE) within 12 to 48 h. For comparison, ticks were fed to engorgement solely on rabbit or mouse for 5 days as controls (naturally engorged on rabbit, NEr, or mouse, NEm). Blood digestion-related gene expression in the midgut and reproductive fitness were compared. Body weight, egg mass weight, egg conversion ratio, and hatchability of eggs did not show any significant differences. We analyzed transcription profiles of selected genes assayed by quantitative RT-PCR and revealed similar patterns of expression between NEr and AE but some differences between NEm and AE or NEm and NEr.

**Conclusions:**

Our results demonstrate that this semi-artificial feeding technique mimics natural feeding processes of ticks and can be utilized as a standardized method to inoculate pathogens, especially *Babesia* protozoa, into *H. longicornis* and possibly other tick species as well.

## Findings

As vectors of pathogens, ticks transmit viruses, rickettsia and protozoan parasites to both animals and humans. Artificial feeding systems are attractive tools for investigating the mechanisms of pathogen transmission as well as for studying the tick-pathogen interactions. First, artificial feeding systems can reduce variation within a given treatment group because the blood meal is supplied from the same donor, which reduces the variation that arises from individual host-tick relationships [1]. Second, an animal experimental model is assumed to have a potent difficulty to control the infection in attached ticks with known numbers of pathogens, because pathogen load in ticks might be affected by the immune system of the hosts targeting tick molecules, such as protective antigen, subolesin [2]. In this model using artificial feeding, the effects of the host’s immune responses against ticks are removed, and pathogens can be introduced into vectors in a controlled manner. So far, artificial feeding techniques have been used to feed a number of tick species of the family Ixodidae, including *Rhipicephalus* spp., *Dermacentor* spp., *Amblyomma* spp., *Hyalomma* spp., and *Ixodes* spp. using capillary tubes or membranes (briefly reviewed in [3]). Recently, Kröber and Guerin [1,4,5] established a method using a silicone membrane to engorge *Ixodes ricinus*. Tajeri and Razmi [3] also attempted to use this membrane for *Hy. anatolicum anatolicum* and *Hy. dromedarii*. These tick species have a long hypostome and fine palps with a wide range of motion and can reasonably be expected to completely penetrate the artificial membrane.

**Figure 1:**
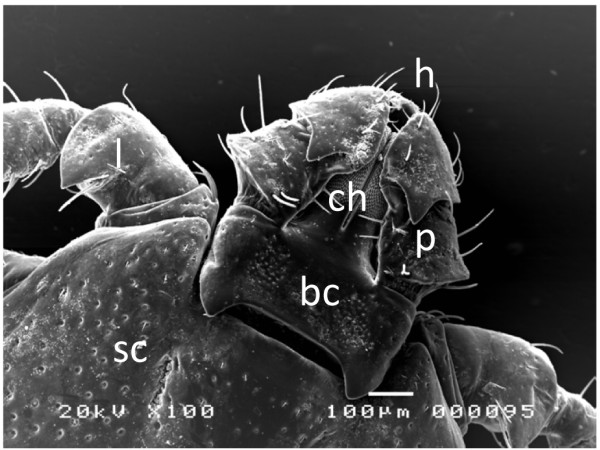
**Capitulum of *H. longicornis*.** Scanning electron micrographs showing the mouthparts of *H. longicornis* from the dorsal aspect. Bar = 100 μm. h, hypostome; ch, chelicera; p, palps; bc, basis capituli; sc, scutum; l, leg.

**Figure 2:**
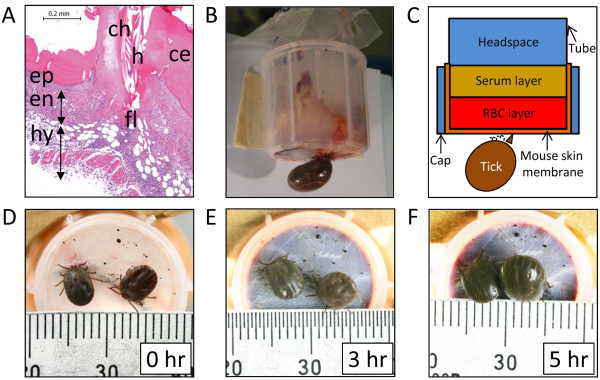
**Mouse-skin membrane-based semi-artificial feeding device.** (**A**) Tick bite lesion of mouse skin used as a membrane in semi-artificial feeding technique. h, hypostome; ch, chelicera; ce, cement; en, endodermis; ep, epidermis; hy, hypodermis; fl, feeding lesion. (HE, Bar: 200 μm) (**B**) Lateral view of the semi-artificial feeding system with a feeding tick. (**C**) Schematic diagram of the system used in this study. (**D-F**) Chronological changes (0, 3, 5 h) of ticks feeding on the semi-artificial feeding system.

On the other hand, *Haemaphysalis longicornis*, another ixodid tick, has a short hypostome and trigonal palps projecting laterally with very limited motion (Figure 1). Additionally, its hypostome and chelicerae are not thrust deeply into the host’s dermis (Figure 2A) [6], therefore, it is quite difficult for *H. longicornis* to feed using capillary tube or silicone membrane-based *in vitro* feeding systems. To overcome these limitations, here, ticks were first fed on a mouse for 4 to 5 days, and then, the skin with the attached ticks was removed from the mouse for construction of a feeding system. This technique is a semi-artificial feeding technique and ticks were fed with supplied rabbit blood, and here, designated as artificially engorged (AE) ticks. To evaluate the physiological effects of this technique, post-engorgement phenotype, including blood feeding (engorgement body weight), reproductive fitness (egg mass weight and egg conversion ratio), and hatchability of eggs to larvae, as well as transcription profiles of selected genes expressed in midgut were compared to those of naturally engorged ticks fed on tick-naïve SPF rabbit (NEr) or mouse (NEm).

### Naturally engorged ticks

The parthenogenetic Okayama strain of the tick *H. longicornis* has been maintained by feeding on rabbits in our laboratory since 1976 [7]. In this study, NEr ticks were prepared according to the usual method described previously [8]. Briefly, 20 adult ticks were placed on the ears of a rabbit to feed. At the beginning of the engorgement period, 9 ticks, which spontaneously detached from the rabbit after 5 days were collected. Of those, 6 randomly selected ticks were subjected to phenotypic analysis, and the remaining 3 ticks were used for transcription analysis. NEm ticks were also prepared according to the method described previously [9]. Briefly, 5 mice (BALB/c, 3 weeks old) were infested with 10 adult ticks (2 ticks per mouse). Six randomly selected NEm ticks were subjected to phenotypic analysis, and the remaining 4 ticks were used for transcription analysis. Ethical approvals of conducting all animal experiments were provided by the Animal Care and Use Committee, National Institute of Animal Health (NIAH, Approval nos. 441 and 578).

### Artificially engorged ticks

To prepare the mouse skin membrane, 5 to 7 adult ticks were allowed to feed on the shaven back of each of four tick-naïve SPF mice (BALB/c, 3 weeks old) following the described method [9]. After 4 to 5 days (at the beginning of the expansion period [10]), a rectangular section (~9 cm^2^) of the mouse skin with the ticks attached was carefully removed from the mouse’s body. Figure 2A shows the Hematoxylin and Eosin (HE)-stained lesion of mouse skin membrane used for the feeding system. Hypodermal layers around the feeding lesion were carefully removed from the skin with sterile tweezers as much as possible. The body of the feeding system (Figure 2B) was constructed using the upper part of a Falcon tube (#352059, Becton, Dickinson and Company, Franklin Lakes, NJ) by cutting the tube at ~3 cm from the top. Then, the roof of the cap of the Falcon tube was removed and the skin membrane with ticks was placed on the mouth of the tube keeping the ticks outside and held tightly in place by applying the cap. Only two ticks were selected and allowed to feed within the area of the skin membrane in this system, and the ticks in excess of two were removed by tweezers and weighed. The mean body weight of the removed ticks (24.9 ± 5.2 mg) was subtracted from the weight of ticks after semi-artificial feeding to estimate weight gain. After construction, the inside of the membrane was washed with sterilized phosphate-buffered saline (PBS) supplemented with 100 units/ml penicillin and 100 μg/ml streptomycin (Life Technologies Corporation, Carlsbad, CA). Then, pre-warmed (30°C) rabbit blood containing 300 μl washed red blood cells (RBC) and 700 μl sterile serum (filtered with a syringe filter; #4652, 0.2 μm, Acrodisc Syringe Filters, Pall Co., Cornwall, UK.) was poured into the device (Figure 2C). To secure a sufficient volume of blood for the duration of tick feeding, we collected blood from a tick-naïve female SPF Japanese white rabbit (3- to 5-months-old). The open end of the tube was covered with a piece of parafilm. All procedures including system construction and blood exchange were performed inside a biosafety cabinet. The system was kept in a humidified chamber with >95% relative humidity at 30°C, and the rabbit blood was changed at every 12 h. When partially fed ticks of the expansion period (4–5 days post-infestation, DPI) were used, fully engorged ticks dropped off within 12 to 48 h of the onset of artificial feeding (Figure 2D-F). In contrast, ticks in the late-growth phase (3 DPI) required feeding on blood for more than 48 h to become engorged (data not shown). These feeding patterns were quite similar to the on-host feeding patterns of ticks described previously [10], suggesting that our *in vitro* feeding device effectively supports the expansion process of ticks and provides a sufficient amount of blood.

### Phenotypic analysis

**Figure 3:**
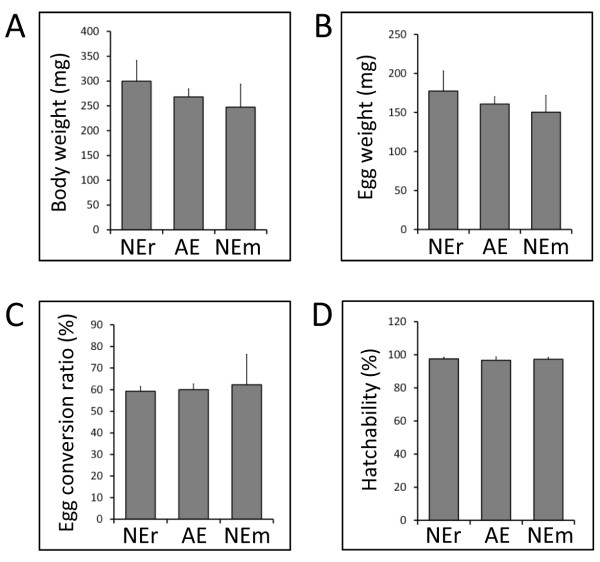
**Phenotypic comparison of ticks fed on different feeding systems.** (**A**) Engorged body weight of NEr (n = 6), AE (n = 4), and NEm (n = 6) ticks. The bars represent mean values and the error bars indicate the standard deviation. (**B**) Total egg mass weight measured at 10 DPO. (**C**) Egg conversion ratio (total egg weight/engorged body weight). (**D**) Comparison of hatchability of eggs (larval counts/total number of larvae and non-hatching eggs) derived from engorged ticks of different feeding systems. The results shown are from a single experiment and are representative of three independent experiments.

Blood feeding, reproductive parameters, and hatching rate of eggs were quantified to investigate the influences of artificial feeding on tick physiological phenotype. After repletion, the body weight gain of AE ticks was approximately 10 times that of their initial body weight. There were no significant differences in engorged body weight between AE and NEr or NEm ticks measured after spontaneous detachment of ticks following full engorgement (Figure 3A). After collection, the engorged ticks were placed separately in small sterile vials and incubated in a humidified chamber with >95% relative humidity at 25°C for egg production. Almost all of the ticks started laying eggs at 4 to 5 days post-engorgement, however, ticks were monitored until 10 days post-oviposition (DPO) at which time point each egg mass obtained from individual ticks was collected separately and weighed. To determine hatching rates, the collected egg mass was placed separately in a paper envelope and incubated under the same conditions described above for 40 days until the eggs hatched. After hatching, larvae were counted manually and hatchability was estimated as described previously [11]. There were no significant differences between AE and NEr or NEm in terms of egg mass weight (Figure 3B), egg conversion ratio (total egg weight/engorged body weight, Figure 3C), or hatching rate (Figure 3D). In addition, larvae derived from the AE, NEr, and NEm lineages of ticks were able to feed normally on rabbit (data not shown).

### Transcription analysis

**Figure 4:**
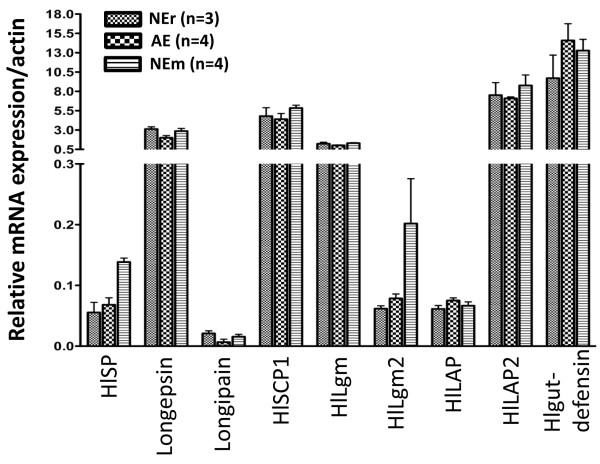
**Comparison of transcriptional patterns of midgut-associated genes.** Quantitative RT-PCR analysis of gene expression is presented relative to the internal standard *β-actin* and error bars represent the standard deviation. The results shown are from a single experiment and are representative of three independent experiments.

We evaluated the effects of artificial feeding on the transcription of eight selected genes related to proteolysis of tick blood digestion in the midgut [12] such as *H. longicornis* serine proteinase *(HlSP)* [GenBank:AB127388] [13], *Longepsin* [GenBank:AB218595] [14], *Longipain* [GenBank:AB255051] [15], *H. longicornis* serine carboxypeptidase 1 (*HlSCP1*) [GenBank:AB287330] [16], *H. longicornis* legumain (*HlLgm*) [GenBank:AB279705] [17], *HlLgm2* [GenBank:AB353127] [18], *H. longicornis* leucine aminopeptidase (*HlLAP*) [GenBank:AB251945] [19], and *HlLAP2* (Hatta and Tsuji, unpublished data). Additionally, we checked one *defensin* gene related to tick innate immunity in the midgut [20], namely, *H. longicornis* midgut defensin (*Hlgut-defensin*) [GenBank: EF432731] to judge the microbial contamination or infection in AE ticks since the transcription of this gene is drastically induced by lipopolysaccharide (LPS) [21]. To do this, the midguts of NEr, AE, and NEm ticks were collected in sterile PBS at 3 days after repletion. Immediately after collection, the midguts were submersed into 3 ml of Buffer RLT (RNeasy Mini Kit, Qiagen, Valencia, CA, USA) supplemented with 30 μl of 2-mercaptoethanol, and thoroughly homogenized by passing the tissue mass through a 20-gauge needle (NN-2038R, Terumo, Tokyo, Japan) fitted to a 5 ml syringe (SS-05LZ, Terumo) five times. Total RNA was isolated from the homogenates according to the manufacturer’s instructions and used to synthesize cDNA (RNA PCR Kit Ver 3.0, Takara Bio INC., Shiga, Japan). Quantitative reverse transcription polymerase chain reaction (qRT-PCR) using SYBR Green I dye (LightCycler FastStart DNA Master SYBR Green I, Roche Diagnostics, Mannheim, Germany) and the primer sets listed in Table 1 was conducted to measure mRNA expression levels, as described previously [22]. Expression of each of the genes was normalized to that of *β-actin* [GenBank:AY254898] based on copy number (Figure 4). As expected, blood digestion-related gene expression patterns were quite similar between AE and NEr ticks, suggesting that the semi-artificial feeding technique does not affect midgut function parameters, such as blood uptake, expansion of midgut size, and blood-digestion. However, a slight difference in expression of two genes (HlSP and HlLgm2) was observed in ticks of the feeding groups AE and NEm or of NEr and NEm, which may be due to the feeding of blood from different hosts, corroborating the findings of others [23].

**Table 1:** Primers for quantitative RT-PCR

**Gene name and primer ID**	**Primer sequences (5′ to 3′)**	
**β-actin**		
HlActin8F1	CCCATCTACGAGGGTTACGCTC	
HlActin9R1	CATCTCCTGCTCGAAGTCCAGG	
**HlSP (serine protease)**		
HlSPEf2	TCCTTCCTCCTGAAGCAG	
HlSPEr2	CGTTCGCTATCCATGGTC	
**Longepsin (aspartic protease)**		
longepsin_f2	CATGAACGGCGTGAAAGTAG	
longepsin_r2	TCCTTGCCTTCCAAAACG	
**Longipain (cysteine protease)**		
longipain_f1	ACCCTGCGACAAGAGCATAC	
longipain_r1	TCCACTTGAATCTGCGTCAC	
**HlSCP1 (serine carboxypeptidase)**		
HlSCP1_f1	TGCTTCAGACTGCATTGACC	
HlSCP1_r1	TTGACCGCAGGTGTCATATC	
**HlLgm (legumain)**		
HLleg1RTf1	CGACGAGCAAATCGTAGTCA	
HLleg1RTr1	ACTTTTCCGCTTCCTCCATT	
**HlLgm2 (legumain)**		
B32G12f1	CCTTCGCAACAAGCTAAAGG	
B32G12r1	TCAGAAGTCCTTCGGTGCTT	
**HlLAP (aminopeptidase)**		
HlLAPf1	CGCTAAGAAGCAGGCTGTCCTA	
HlLAPr1	TCAGACCGTAGAAAACTCTGGAC	
**HlLAP2 (aminopeptidase)**		
HlLAP2f2	AAGGCTCTTCACGAAGTGGA	
HlLAP2r1	TGGTCGACACCTCGAACATA	
**Hlgut-defensin (anti-microbial peptide)**		
EF132731 F	GGGACTTTTACTGGCTTTCCTG	
EF132731 R	ACACGCCCTTTCATCGAAC	

Even though a biosafety cabinet was used throughout the study during construction and manipulation of the feeding device, the possibility of microbial contamination in the blood cannot be completely neglected since we did not use antibiotics in the supplied blood. Nevertheless, similar expression patterns of the *Hlgut-defensin* gene among AE, NEr, and NEm ticks indicate that the feeding system was quite free from bacterial contamination. However, to increase the certainty of avoiding bacterial contamination, it would be better to apply antibiotics to this feeding system for other applications.

## Conclusions

In conclusion, our findings suggest that the semi-artificial feeding technique for *H. longicornis* is very effective and may be used for all tick species, especially those with short hypostomes that do not penetrate deeply in the dermal layer of the host, such as *Rhipicephalus* ticks [24]. Additionally, the technique is quite simple and cost effective since it does not require thinner artificial membrane [3], odorant (cow hair extracts), and/or feeding stimuli (adenosine triphosphate, ATP) [1,4,5] to enhance tick attachment. Although it is necessary to sacrifice mice in order to construct the device, the technique bears great promise for conducting *in vitro* assays, including the inoculation of pathogens, especially *Babesia* protozoa, based on results reported by Callow [25] that *Rhipicephalus* (*Boophilus*) *microplus* tick infection with one of the tick-borne bovine pathogens, *Babesia bigemina*, takes place during the rapid phase of feeding corresponding to the final stage (last 24 h) of the blood feeding. We expect that this technique will be useful in studies of tick physiology, tick-pathogen interactions, and tick-host interactions regarding novel tick genes that respond to the host defense mechanisms such as coagulation, inflammation, and immune responses.

## Abbreviations

AE: Artificially engorged; ATP: Adenosine triphosphate; DPI: Days post-infestation; DPO: Days post-oviposition; HE: Hematoxylin and eosin; HlSP: *H. longicornis* serine proteinase; HlSCP1: *H. longicornis* serine carboxypeptidase 1; HlLgm: *H. longicornis* legumain; HlLAP: *H. longicornis* leucine aminopeptidase; NE: Naturally engorged; NIAH: National Institute of Animal Health; PBS: Phosphate-buffered saline; RBC: Red blood cells; RT-PCR: Reverse transcription polymerase chain reaction.

## Competing interests

The authors declare that they have no competing interests.

## Authors’ contributions

TH, NT, and KF were involved in the design of the experiments. TM, MAA and MM carried out the rabbit experiment. A and KY carried out the mouse experiment. The manuscript was prepared by TH, MKI and NT. All authors actively contributed to the interpretation of the findings, and read and approved the final manuscript.
